# Upregulated Expression of Profilin1 on Dendritic Cells in Patients With Severe Aplastic Anemia

**DOI:** 10.3389/fimmu.2021.631954

**Published:** 2021-06-16

**Authors:** Hong Yu, Yang Zhao, Xiaofeng Pan, Chunyan Liu, Rong Fu

**Affiliations:** Department of Hematology, Tianjin Medical University General Hospital, Tianjin, China

**Keywords:** severe aplastic anemia, myeloid dendritic cells, profilin1, antigen presenting cells, bone marrow failure

## Abstract

Severe aplastic anemia (SAA) is a life-threatening form of bone marrow failure that is associated with very high mortality. Dendritic cells (DCs) are antigen presenting cells (APCs) with powerful movement ability, which is an important factor affecting immune function. The expression of profilin1 (Pfn1) plays an important role in the regulation of cell movement ability. We detected the expression of Pfn1 mRNA in the bone marrow (BM) myeloid dendritic cells (mDCs) from patients with SAA using RT-PCR. Next, we examined Pfn1 expression on mDCs using flow cytometry (FCM). We also assessed the relationship between Pfn1 expression and cytokine levels. Our data showed increased Pfn1 mRNA expression in patients with SAA. The expression of Pfn1 in BM mDCs increased in SAA patients. The expression of Pfn1 on mDCs and cytokines (TNF-α and IFN-γ) were positively correlated in the serum of untreated patients with SAA. Taken together, we found that the expression of Pfn1 on mDCs of SAA patients increased, which may affect the function of mDCs. Profilin 1 may be involved in the immunopathogenesis of SAA.

## Introduction

Severe aplastic anemia (SAA) is a life-threatening form of bone marrow failure which, if untreated, is associated with very high mortality (1). Acquired SAA is due to autoimmune destruction of multipotent hematopoietic stem cells dominated by abnormal cellular immunity (2, 3). Our previous research found that the number of myeloid dendritic cells (mDCs) in SAA patients was significantly higher than that in the normal control group, but there was no significant difference in the number of lymphoid dendritic cells (pDCs) (4–6). The ratio of mDCs/pDCs in SAA patients significantly increased, and mDCs highly expressed the costimulatory molecule CD86. After immunosuppressive therapy (IST), the number of mDCs and the ratio of mDCs/pDCs in SAA patients decreased significantly (6). Activated mDCs can secrete a large amount cytokines (e.g. IL-12), which can activate Th1 cells. IL-12 can induce the expression of T-bet, a transcription factor related to Th1 polarization in Th0 cells, and promotes the generation of Th1 cells. Our study confirmed that Th1/Th2 balance shifted to Th1 in SAA patients. The number of Th1 cells in treatment-naive SAA patients increased significantly, and the cytokines (e.g. INF-γ, TNF-α and IL-2) associated with Th1 cells increased significantly (7). The above factors together lead to excessive activation of cytotoxic T lymphocytes (CTLs). CD8 + T effector cells damage the bone marrow hematopoietic cells of patients through perforin, granzyme B pathway, Fas/FasL pathway and TNF-β pathway (8).

Dendritic cells (DCs) are antigen presenting cells (APCs) with powerful movement ability, which is an important factor affecting its immune function. Apoptosis, migration and movement of DCs all affect the production of mDCs activation effector T cells (9). The movement of cells is a complex process involved with multiple proteins in regulation. When the cells do directional migration, the dissociation and aggregation of actin occur simultaneously, causing the cell morphology to change toward the stimulated side. Cofilin, Fascin, profilin 1 and other cytoskeletal binding proteins are involved in the regulation of this process (10). The family of profilin, the first actin-binding proteins to be characterized, play important roles in actin dynamics (11). Mammalian profilin family consists of four members: profilin 1, which is widely expressed, and profilin 2–4, which have a conserved expression pattern (11, 12). Previous studies have indicated crucial roles for profilins in many cellular processes, such as membrane trafficking, small GTPase signaling and nuclear activities, as well as tumor formation and neurological diseases. Actin is the main ligand of profilins, which indicates its role in actin-driven cellular motility. The expression level of profilin1 in cells determines the regulation of cell movement ability. Additionally, recent research has shown that Pfn1 has a strongly immunomodulatory effect on dendritic cells. However, the relationship between Pfn1 and mDCs in the context of SAA remains unclear. In this study, we aimed to investigate the role of Pfn1 in mDCs activation in SAA patients and to provide data to support a potential mechanism of mDCs activation and the immune process in this population.

## Materials and Methods

### Patient Description

Thirty-six patients diagnosed with SAA including 16 males and 20 females with a median age of 28 years (range,21–65 years), was admitted to hematology department, Tianjin Medical University General Hospital from June 2020 to November 2020. The diagnosis of AA and disease severity were defined according to the International AA Study Group Criteria. Severe AA (SAA) is defined as hypocellular bone marrow with two out of three of the following parameters: neutrophil count <0.5 × 10^9^/L, platelet count <20 × 10^9^/L and reticulocyte count <20 × 10^9^/L (13). The patients’ basic information and clinical characteristics are provided in **Table 1**. The clinical characteristics of SAA patients was significantly different from that of R-SAA patients (P < 0.01).

**Table 1:** The clinical characteristics of all the patients.

Diagnosis	Untreated SAA	R-SAA
Number	16	20
Age (years)	35 (11-65)	30 (12-73)
ANC (*10^9^/L)	0.48 ± 0.36^**^	3.87 ± 1.52
Hb (g/L)	77.64 ± 8.94^**^	137.1 ± 18.40
PLT (*10^9^/L)	38.08 ± 14.44^**^	127.3 ± 62.28
Ret%	0.39 ± 0.19^**^	2.71 ± 1.88
Therapy	Not previously treated except for transfusions	IST

Values are expressed as mean ± SD. Age was mid (min, max). ANC absolute neutrophil count, Hb hemoglobin, PLT platelet, Ret reticulocyte **p < 0.01.

The normal controls included 9 males and 9 females with a median age of 27 years (range from 24 to 58 years). BM samples were taken from their postoperative discarded ribs. Written informed consent was taken from each patient and the study was in accordance with the Declaration of Helsinki. This study was approved by the Ethics Committee of Tianjin Medical University General Hospital.

### mDCs Culture *In Vitro*

BM mononuclear cells (BMMNCs) were extracted from patients with SAA and healthy controls using lymphocyte separation fluid (Solarbio Science & Technology) by density gradient centrifugation. BMMNCs of each subject were plated separately at a density of 2x10^6^ cells/ml in RPMI-1640 (Gibco BRL, Grand Island, NY, USA) complete medium containing 10% fetal bovine serum (Gibco BRL, Grand Island, NY, USA) and 1% mycillin and incubated for 2 h at 37°C in an atmosphere containing 5% CO2. Non-adherent cells were removed. The remaining cells were cultured in RPMI-1640 complete medium containing 10% fetal bovine serum, 1% mycillin, 100 ng/ml rhGM-CSF (cat.no. 10015-HNAH, Sino Biological, Beijing, China) and 40 ng/ml rhIL-4. The culture conditions were 37°C and 5% CO_2_. Media and cytokines were replaced every two days. On day 6, rhTNF-α (1,000 μg/ml) was added to mature the mDCs for 24 h. Suspended mature mDCs in the culture supernatant were then collected on day 7.

### Real-Time Polymerase Chain Reaction

For mDCs Pfn1 mRNA expression analysis, total RNA was extracted using Trizol (Invitrogen, Carlsbad, CA, USA). BMMNCs of patients with SAA and controls were lysed using TRIzol reagent. RNA was reverse transcribed using the complementary (c)DNA Synthesis Kit (Tiangen). qPCR was performed on the BIORAD iQ5 system (Bio-Rad Laboratories, Inc.). GAPDH was used as a housekeeping gene for standardizing targeted mRNA expression. Primers for amplifying GAPDH and profilin1 are listed in **Table 2**. The relative expression level of the gene of interest was calculated using the 2^-ΔΔCT^ method (14).

**Table 2:** Primers for real-time quantitative PCR detection.

Primer	Sequence (5′ to 3′)
Profilin 1	Forward: 5'- CGCCTACATCGACAACCTCATGG -3'
	Reverse: 5'- AGCTGGCGTGATGTTGACGAAC-3'
GAPDH	Forward: 5'-TTCCACCCATGGCAAATTCC-3'
	Reverse: 5'-AGGCCATGCCAGTGAGCTTC-3'

### Flow Cytometry

Peripheral blood samples (100μl) anticoagulated by EDTA were stained with HLADR-PerCP, CD11c-PE, profilin 1 -APC and the isotype controls in separated tubes (BD PharMingen) for 15 min at 4°C in the dark, followed by erythrocyte lysis using 2mL erythrocyte lytic solution (BD PharMingen). Finally, 50,000 cells were acquired on a Beckman flow cytometry and analyzed by CytExpert software.

### Cytokine Detection

A cytokine detection kit (Human Th1/Th2 subsets detection kit)was used for this analysis (cat. no. P02080100013; Saijishengwu). The venous blood samples were collected in EDTA anticoagulation tubes and centrifuged at 1,000 x g for 20 min for later use. Standards were configured with the following concentrations: 10, 20, 40, 80, 156, 312, 625, 1,250, 2,500 and 5,000 pg/ml. The captured microsphere mixture was centrifuged at 200 x g for 5 min at room temperature. The supernatant was aspirated, the same volume of microsphere buffer as the aspirated supernatant was added, and following thorough mixing, the sample was incubated for 30 min at room temperature. Subsequently, 25 µl of the solution was added to each experimental tube and the sample was vortexed. A total of 25 µl of standard product and 25 µl of the sample to be tested was added in the same sample tube. Subsequently, 25 µl of the fluorescence detection reagent was added to each experimental tube and samples were thoroughly mixed and incubated at room temperature for 25 min in the dark. Finally, 1 ml PBS was added to each experimental tube, which was then centrifuged at 200x g for 5 min at room temperature. The supernatant was aspirated and 100 µl PBS was added for fluorescence detection on the flow cytometer (BD Biosciences; FACSCanto II). This was according to the manufacturer’s instructions.

### Statistical Analysis

Statistical analyses were performed using SPSS 22.0 software (IBM Corp.). Data analyses were performed with GraphPad Prism 5.0 software (GraphPad Software, Inc.). The normality of the distribution was proven using a Kolmogorov-Smirnov test. The mean ± standard deviation was used to represent normally distributed data. Non normal distribution data are represented by median (interquartile interval). Comparisons between two independent samples were performed using the t-test. For correlation tests, Spearman’s rank correlation was used. P<0.05 was considered to indicate a statistically significant difference.

## Results

### The Expression of Profilin 1 on mDCs in SAA Patients and Normal Controls

**Figure 1:**
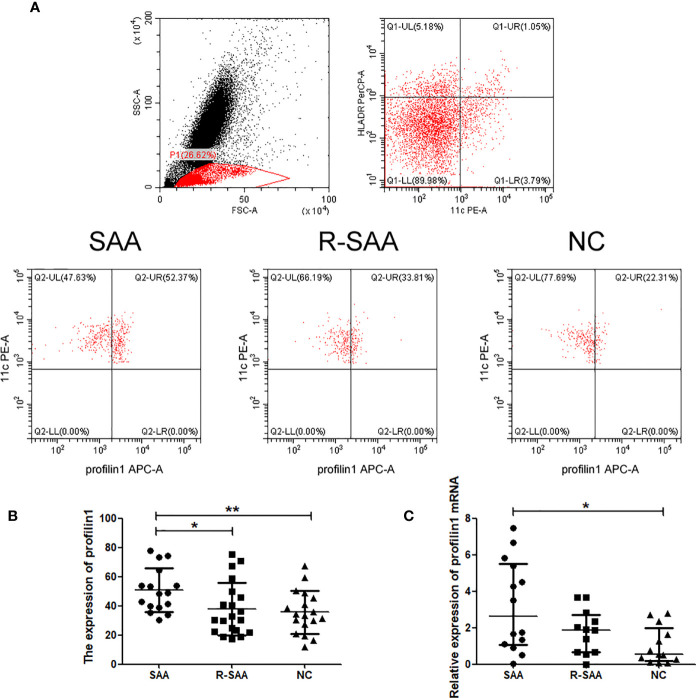
The expression of profilin 1 on mDCs in SAA patients and normal controls **(A)** Expression assessed using flow cytometry. **(B)** Profilin 1 expression on CD11C+ HLA-DR+ cells in patients with SAA and normal controls. **(C)** Relative expression of profilin1 mRNA in mDCs determined using reverse transcription-quantitative PCR. ^*^p < 0.05, ^**^p < 0.01.

Representative flow cytometry of profilin 1 in mDCs is shown in **Figure 1A**. The expression of profilin 1 among mDCs of untreated patients with SAA (50.69 ± 14.93%) was significantly higher than that of the remission patients and controls (37.80 ± 18.15%, 35.59 ± 14.68%, respectively) (P < 0.05, P < 0.01). There were no significant differences in the percentages between the remission patients with SAA and controls (P > 0.05) (**Figure 1B**).

### Profilin 1 mRNA Expression on mDCs in SAA Patients and Normal Controls

**Figure 2:**
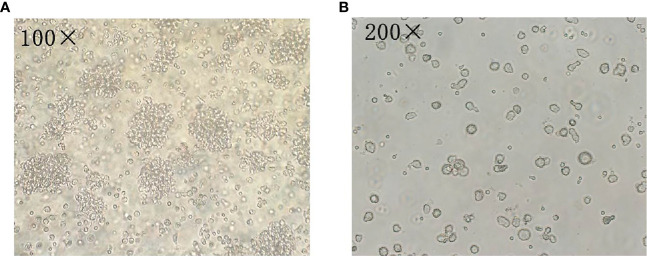
Analysis of mDCs using microscopy. **(A)** The mDCs was observed by microscope after 7 days of culture (100×). **(B)** The mDCs was observed by microscope after 7 days of culture (200×).

After sorting, the purity of mDCs was more than 90% (**Figure 2**). Furthermore, we detected Pfn1 mRNA expression in mDCs isolated from bone marrow in SAA patients and normal controls. Pfn1 mRNA expression on mDCs in untreated SAA group was significantly higher than that in normal control group [2.623 (1.051-5.496) vs 0.5586 (0.1881-1.991), P <0.05]. The expression of Pfn1 mRNA in patients with SAA remission patients was higher than that in normal control group, but there was no significant difference [1.879 (0.6748- 2.709) vs 0.5586 (0.1881-1.991), P > 0.05] (**Figure 1C**).

### Frequency of Profilin 1 Was Associated Closely With the Number of Immune Cells and the Concentration of Cytokine Levels in Patients With SAA

**Figure 3:**
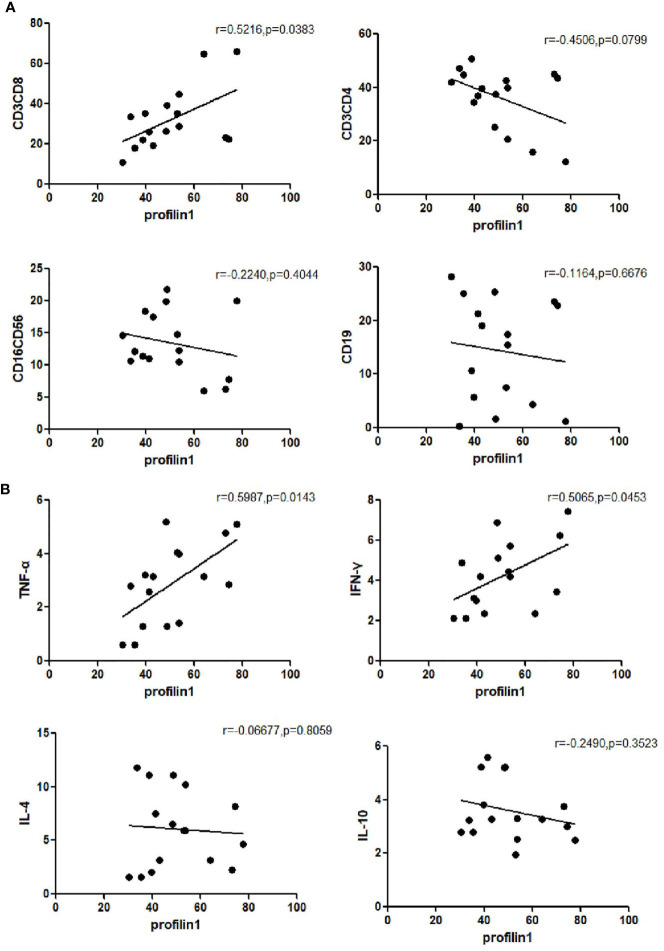
Frequency of profilin 1 was associated closely with the number of immune cells **(A)** and the concentration of cytokine levels **(B)** in patients with untreated SAA patients.

The expression of Pfn1 on mDCs and the number of CD3+CD8+ cells (r=0.5216, *P*=0.0383) were positively correlated in the untreated patients with SAA. There was no significant correlation between the expression of Pfn1 on mDCs and the number of CD3+CD4+ cells (r=-0.4506, *P*=0.0799), CD16+CD56+ cells (r=-0.2240, *P*=0.4044) and CD19+ cells (r =-0.1164, *P*=0.6676) in treatment naive patients with SAA (**Figure 3A**).

The expression of Pfn1 on mDCs and the concentration of cytokine TNF-α (r=0.5987, *P*=0.0143) and the concentration of IFN-γ (r=0.5065, *P*=0.0453) were positively correlated in the serum of untreated patients with SAA. There was no significant correlation between the expression of Pfn1 and the concentration of cytokine IL-4 (r=-0.06677, *P*=0.8059) and IL-10 (r=-0.2490, *P*=0.3523) in untreated patients with SAA (**Figure 3B**). We detected the concentration of cytokines in patients with SAA in remission, and detected the correlation with the expression of profilin1 on mDC, but there was no statistical significance.

## Discussion

In the course of exploring the pathogenesis of SAA, it was found that the number of mDCs in SAA patients was significantly higher than that of normal people, and the ratio of mDC/pDC was significantly increased (15–18). The expression of costimulatory molecule CD86 on the surface of mDCs was significantly increased (5). The expression of costimulatory molecule CD86 on the surface of mDCs was significantly increased. These results suggest that mDCs is in a state of hyperfunction and its antigen presentation ability is enhanced. The activated mDCs secretes a large amount of IL-12 and T-bet, which can promote the polarization of Th0 to Th1 and make Th1/Th2 imbalance. Co-culture of mDCs and lymphocytes from SAA patients *in vitro* suggests that mDCs stimulates lymphocytes proliferation. mDCs is the most powerful APCs discovered so far. It matures gradually during the process of uptake and processing of antigens. It highly expresses MHC class I and II molecules, costimulatory molecules CD80, CD86, ICAM-1, CD83, and inflammatory cells factors (e.g. IL-12 and IL-18), which can promote the polarization of Th0 to Th1 and then initiate cellular immunity. Thus, it plays a very important role in the primary link of immune response-antigen recognition and presentation. Furthermore, mDCs is the core in the activation of the immune response against pathogens and the regulation of peripheral tolerance to self-antigens. It can ingest, process and present antigens and stimulate the activation and proliferation of naive T cells. It is both an initiator and a regulator. The central link in regulating and maintaining a specific immune response.

The extracellular uptake, maturation, migration, presentation, proliferation and other physiological activities of mDCs are closely related to its cytoskeleton, which requires actin remodeling to drive the formation of silk feet or plate feet, and cytoplasmic extension of the cytoskeleton rearrangement. The cytoskeleton is composed of microfilaments, intermediate fibers and microtubules. Cell movement depends on actin polymerization, and the dynamic regulation of actin depends on a variety of actin-binding proteins (ABPs) to regulate monomeric actin (G-actin) and filamentous actin (F- actin) (19). Mainly polymerized from monomeric actin to form different types of fibrous actin, microfilaments participate in cell movement. In DCs, this structure is of great importance. It not only affects the movement of DCs, but can even affects the interaction between mDCs and T cells. The movement of cells depends on the continuous dissociation and polymerization of F-actin, which is completed with the participation of actin binding protein. So far, nearly 200 binding proteins involved in actin assembly have been discovered, including cofilin and integrin complexes, ARP2/3, formins, profilin family, capping protein, etc (20, 21).

Pfn1 is the first actin-binding protein to be discovered, which can bind to the ADP-actin monomer released from the fibrous end of F-actin, convert it into ATP-actin monomer and load it into the extended end of F-actin (22). The profilin family discovered so far includes profilin1, profilin2, and profilin3, among which profilin 1 has the highest affinity for actin protein. Changes in the expression of Pfn1 can regulate the expression levels of proteins involved in movement, proliferation and apoptosis, promoting actin polymerization and changes in cytoskeleton dynamics, and have become a key regulator of cell function (23, 24). Therefore, cells regulate their motility through the expression of Pfn1. The integrity of the mDCs cytoskeleton plays a vital role in the activation of T cells (25).

It was found that the expression of Pfn1 in DCs of SAA patients was significantly increased, and positively correlated with number of CD3+CD8+ cells in peripheral blood and TNF-α and IFN-γ in peripheral blood serum. The expression of Pfn1 can reflect the disease severity of SAA patients. Profilin 1 may become an indicator to predict the prognosis. This is the first report of structural proteins and SAA in the pathogenesis of AA. In the future, we will further study the related pathways and mechanisms to find new targets for the treatment of SAA.

## Data Availability Statement

The original contributions presented in the study are included in the article/supplementary material. Further inquiries can be directed to the corresponding author.

## Ethics Statement

The study was in compliance with the Declaration of Helsinki and was approved by the Ethics Committee of Tianjin Medical University General Hospital (Tianjin, China). Written informed consent was obtained from all participants. Written informed consent to participate in this study was provided by the participants’ legal guardian/next of kin. Written informed consent was obtained from the individual(s), and minor(s)’ legal guardian/next of kin, for the publication of any potentially identifiable images or data included in this article.

## Author Contributions

RF designed the study and revised the manuscript. HY and YZ performed experiments, analyzed data, and wrote the initial draft of the manuscript. XP and CL contributed to the experiments and the collection of patients’ features. All authors contributed to the article and approved the submitted article.

## Funding

This work was supported by the National Natural Science Foundation of China (grant nos. 81970116, 81970115, 81870101 and 81500101) and the Tianjin Municipal Natural Science Foundation (grant nos. 18JCYBJC91700 and 18ZXDBSY00140).

## Conflict of Interest

The authors declare that the research was conducted in the absence of any commercial or financial relationships that could be construed as a potential conflict of interest.
